# Mortality Rates Among Hospitalized Patients With COVID-19 Infection Treated With Tocilizumab and Corticosteroids: A Bayesian Reanalysis of a Previous Meta-analysis

**DOI:** 10.1001/jamanetworkopen.2022.0548

**Published:** 2022-02-28

**Authors:** Arthur M. Albuquerque, Lucas Tramujas, Lorenzo R. Sewanan, Donald R. Williams, James M. Brophy

**Affiliations:** 1 School of Medicine, Universidade Federal do Rio de Janeiro, Rio de Janeiro, Brazil; 2 HCor Research Institute, Sao Paulo, Brazil; 3 Department of Internal Medicine, Columbia University, New York, New York; 4 Department of Psychology, University of California, Davis; 5 McGill Health University Center, Montreal, Québec, Canada

## Abstract

**Importance:**

A World Health Organization (WHO) meta-analysis found that tocilizumab was associated with reduced mortality in hospitalized patients with COVID-19. However, uncertainty remains concerning the magnitude of tocilizumab’s benefits and whether its association with mortality benefit is similar across respiratory subgroups.

**Objective:**

To use bayesian methods to assess the magnitude of mortality benefit associated with tocilizumab and the differences between respiratory support subgroups in hospitalized patients with COVID-19.

**Design, Setting, and Participants:**

A bayesian hierarchical reanalysis of the WHO meta-analysis of tocilizumab studies published in 2020 and 2021 was performed. Main results were estimated using weakly informative priors to exert little influence on the observed data. The robustness of these results was evaluated using vague and informative priors. The studies featured in the meta-analysis were randomized clinical tocilizumab trials of hospitalized patients with COVID-19. Only patients receiving corticosteroids were included.

**Interventions:**

Usual care plus tocilizumab in comparison with usual care or placebo.

**Main Outcomes and Measures:**

All-cause mortality at 28 days after randomization.

**Results:**

Among the 5339 patients included in this analysis, most were men, with mean ages between 56 and 66 years. There were 2117 patients receiving simple oxygen only, 2505 receiving noninvasive ventilation (NIV), and 717 receiving invasive mechanical ventilation (IMV) in 15 studies from multiple countries and continents. Assuming weakly informative priors, the overall odds ratios (ORs) for survival were 0.70 (95% credible interval [CrI], 0.50-0.91) for patients receiving simple oxygen only, 0.81 (95% CrI, 0.63-1.03) for patients receiving NIV, and 0.89 (95% CrI, 0.61-1.22) for patients receiving IMV, respectively. The posterior probabilities of any benefit (OR <1) were notably different between patients receiving simple oxygen only (98.9%), NIV (95.5%), and IMV (75.4%). The posterior probabilities of a clinically meaningful association (absolute mortality risk difference >1%) were greater than 95% in patients receiving simple oxygen only and greater than 90% in patients receiving NIV. In contrast, the posterior probability of this clinically meaningful association was only approximately 67% in patients receiving IMV. The probabilities of tocilizumab superiority in the simple oxygen only subgroup compared with the NIV and IMV subgroups were 85% and 90%, respectively. Predictive intervals highlighted that only 72.1% of future tocilizumab IMV studies would show benefit. The conclusions did not change with different prior distributions.

**Conclusions and Relevance:**

In this bayesian reanalysis of a previous meta-analysis of 15 studies of hospitalized patients with COVID-19 treated with tocilizumab and corticosteroids, use of simple oxygen only and NIV was associated with a probability of a clinically meaningful mortality benefit from tocilizumab. Future research should clarify whether patients receiving IMV also benefit from tocilizumab.

## Key Points

**Question:**

Can bayesian methods clarify the uncertainty around tocilizumab’s association with mortality benefit in subgroups of hospitalized patients with COVID-19 receiving corticosteroids?

**Findings:**

In this bayesian reanalysis of a previous meta-analysis of 15 randomized clinical trials comprising 5339 hospitalized patients with COVID-19 treated with tocilizumab and corticosteroids, those receiving simple oxygen only or noninvasive ventilation were associated with a clinically meaningful mortality benefit. In contrast, for those receiving invasive mechanical ventilation, an association with benefit was uncertain.

**Meaning:**

This study’s findings indicate that further research is needed to assess the association between mortality benefit or risk in patients with COVID-19 receiving invasive mechanical ventilation and treated with tocilizumab and corticosteroids.

## Teaser

This bayesian reanalysis of a previous meta-analysis of 15 randomized clinical trials evaluates whether treatment with tocilizumab was associated with meaningful mortality benefit among hospitalized patients with COVID-19.

## Introduction

Randomized clinical trials (RCTs) on tocilizumab, an interleukin 6 antagonist, have shown mixed results compared with control or usual care in hospitalized patients with COVID-19 as analyzed in 2021 by the World Health Organization (WHO) Rapid Evidence Appraisal for COVID-19 Therapies (REACT) Working Group.^1^ The authors reported that the association with lower 28-day all-cause mortality “…was more marked among patients who did not require invasive mechanical ventilation (IMV) at randomization.”^1^^(p515)^ However, an exact quantification of tocilizumab’s clinical benefits and associated uncertainties in this or any of the other respiratory subgroups was not provided. Despite this absence, guidelines now suggest the use of tocilizumab in patients with either severe or critical COVID-19 independent of their respiratory status.^2^

Whether additional insights into this evidence base are available by the application of bayesian analytical methods is unknown.^3,4,5^ In brief, bayesian analyses can fully embrace uncertainty and directly answer the clinical question of interest: what is the probability of tocilizumab’s benefit in each subgroup?^6^ Moreover, one could also estimate the probability that tocilizumab’s association with a mortality benefit is different between subgroups. These features can contribute to a more thorough understanding of tocilizumab’s role, avoiding the “nullism” and “dichotomania” inherent with conventional null hypothesis significance testing.^7^

Therefore, we reanalyzed the WHO’s tocilizumab data using bayesian methods, with the primary goal of providing a further appreciation of the results and the reliability of specific subgroup conclusions.

## Methods

This bayesian analysis of a previous meta-analysis was performed in accordance with the Preferred Items for Systematic Reviews and Meta-analyses (PRISMA) guideline.^8^ This study was exempt from obtaining formal institutional review board approval from the McGill University Health Center and the requirement to obtain informed patient consent because it is secondary research of publicly available data sets.

### Data

We included all RCTs collected by the WHO REACT Working Group regardless of publication status or risk of bias. The primary outcome was 28-day total mortality. We added 0.5 events to treatment arms to include studies with no events in one of the arms. Two authors (A.M.A. and L.T.) independently extracted the number of deaths and total patients directly from the WHO REACT Working Group supplementary figures. Only patients receiving corticosteroids were included. In case of any discrepancy, both authors discussed and reached a consensus.

### Meta-analysis

We used a random-effects meta-analysis to perform unconditional inferences beyond the included studies.^9^ Random-effects meta-analysis assumes a distribution of studies, allowing inferences not only on this collection of studies but also on studies that may not have been included in this work.^9^ Therefore, our inferences regard the mean effect of tocilizumab from this hypothetical population of studies,^10^ ie, not exclusively conditioned to the included studies as in a fixed-effect model.

We analyzed tocilizumab’s association with mortality as a function of baseline respiratory disease severity. We included 3 subgroups, defined by the WHO REACT Working Group as supplemental oxygen therapy (oxygen flow rate ≤15 L/min by face mask or nasal cannula) (hereafter, simple oxygen only), noninvasive ventilation (NIV; oxygen flow rate >15 L/min, high-flow nasal cannula, and continuous positive airway pressure), or patients receiving IMV or extracorporeal membrane oxygenation at baseline. We assumed the variability in study outcomes was the same in every subgroup (common between-study heterogeneity).

### Bayesian Analysis

We applied the bayesian framework, which updates prior beliefs with the current data to form a posterior distribution.^6^ In brief, we implemented priors that cover plausible values for all parameters, assigning limited density to impossible values and thereby exerting little influence on the results (hereafter, known as weakly informative priors).^11,12^ To check whether this choice of priors meaningfully affected our results or our conclusions, we also fitted models using vague or informative priors, defined as approximately uniform or centered at the null value with small dispersion, respectively.^13^ Full details about our model and priors can be found in the eMethods of the Supplement.

Bayesian random-effects meta-analysis yields marginal posterior distributions for the treatment associations and between-study heterogeneity. We used medians and 95% highest-density intervals (hereafter, credible intervals [CrIs]) to describe these distributions, defined as the narrowest interval containing 95% of the probability density function.^14^ Our primary estimands are expressed as odds ratios (ORs) and focused on the calculation of posterior probabilities of any benefit (OR <1) and of meaningful clinical association (arbitrarily defined as OR <0.9, ie, at least a 10% odds reduction in outcomes). We also derived the risk difference from the OR across multiple plausible control mortality risks, further explained in the eMethods in the Supplement.^15,16^ In this case, we defined the association with meaningful clinical benefit as a 1% risk difference.

To compare the marginal posterior distributions of each respiratory support subgroup, we generated the ratio of odds ratios (RORs) of each possible comparison: simple oxygen only vs NIV, simple oxygen only vs IMV, and NIV vs IMV. We then calculated the posterior probability of superiority for each comparison (ROR >1).

### Between-Study Heterogeneity and Prediction

By accounting for both within-study and between-study variation, random-effects meta-analysis can better incorporate overall heterogeneity.^6,12^ In the bayesian framework, one acknowledges the prior uncertainty in the heterogeneity parameter and can estimate the posterior distribution of the between-study heterogeneity. We assessed the posterior distribution of the between-study standard deviation (τ, a proxy for heterogeneity) by calculating the posterior probabilities of low, “reasonable,” and “fairly high” heterogeneity (eMethods in the Supplement).^6^

Further, the posterior predictive distribution can better explore the effects of between-study heterogeneity. This distribution allows inference about “…what we would expect to see in a new study population that is exchangeable with the studies included in our meta-analysis.”^17^ The posterior predictive distribution is vital to inform probable values for the true treatment association in future settings, which is valuable for power calculations or prior distribution elicitation in future RCTs.^6,18^ The posterior predictive distribution for each subgroup incorporates the uncertainty both in the overall effect size and the between-study standard deviation parameters and generates new trial population parameter estimates for each subgroup, independent of sample size and other population characteristics.^19^

### Predictive Analysis to Confirm Tocilizumab’s Association With Mortality Reduction

The posterior predictive distribution is useful to predict future settings. However, it does not allow one to directly estimate how many patients a next future trial would have to include to confirm the association of tocilizumab with mortality benefit. To assess future sample size requirements, we generated 6 RCTs of different sample sizes (from 200 to 4000 total patients) comparing tocilizumab and control in patients receiving IMV,^20^ assuming an OR of 0.77 (WHO REACT Working Group mean result for tocilizumab- and corticosteroids-treated patients).^1^ Next, using bayesian normal conjugate analyses, we updated our current knowledge (the meta-analysis results) with these generated RCTs (likelihoods) to form 6 separate posterior distributions. Further explanations about these calculations can be found in the eMethods in the Supplement.

### Model Diagnostics and Code

Meta-analyses and posterior predictive results were conducted using Stan through the R package brms (the R Foundation).^21,22^ Four Markov chains were implemented with an initial warm-up phase of 2000 iterations, followed by 4000 iterations. We followed the When to Worry and How to Avoid the Misuse of Bayesian Statistics Checklist^11,23,24^ for checking details about our analysis, confirming the convergence and adequate sampling of the models. All analyses were conducted in R, version 4.0.4 (R Environment).

## Results

### Studies

Among the 5339 patients included in this analysis, most were men, with mean ages between 56 and 66 years. There were a total of 2117 patients receiving simple oxygen only, 2505 receiving NIV, and 717 receiving IMV in 15 studies from multiple countries and continents.^1,25,26,27,28,29,30,31,32^ Table 1 shows the number of deaths and total patients in each RCT reported in the WHO Working Group publication and included in this reanalysis. As expected, smaller studies showed wider confidence intervals, and these studies were more likely to be unpublished studies. eTable 1 in the Supplement shows general demographic information and a more detailed assessment of sample sizes per trial and subgroup.

**Table 1.** Included Studies

Subgroup/source	Tocilizumab		Control		Odds ratio (95% CI)
	Events	Total	Events	Total	
**Simple oxygen only**					
Stone et al,^25^ BACC Bay, 2020	0	3	0	1	0.43 (0.01-33.60)
COVIDOSE2-SS-A (UNP)	0	6	1	1	0.03 (0.00-1.90)
Hermine et al,^26^ CORIMUNO-TOCI-1, 2021	1	10	3	12	0.33 (0.03-3.84)
Rosas et al,^27^ COVACTA, 2021	1	11	1	12	1.10 (0.06-20.01)
Rosas et al,^29^ REMDACTA, 2021	1	14	0	2	0.56 (0.02-17.92)
Veiga et al,^30^ TOCIBRAS, 2021	1	20	0	14	2.23 (0.08-58.81)
COV-AID (UNP)	1	25	0	21	2.63 (0.10-68.08)
PreToVid (UNP)	12	116	19	118	0.60 (0.28-1.30)
Salama et al,^28^ EMPACTA, 2021	11	128	4	72	1.60 (0.49-5.22)
Abani et al,^31^ RECOVERY, 2021	125	766	173	765	0.67 (0.52-0.86)
**Noninvasive ventilation**					
CORIMUNO-TOCI-ICU (UNP)	1	2	1	1	0.33 (0.01-16.8)
Veiga et al,^30^ TOCIBRAS, 2021	4	5	3	15	16.00 (1.27-200.93)
ImmCOVA (UNP)	2	11	2	18	1.78 (0.21-14.86)
HMO-020-0224 (UNP)	6	15	1	4	2.00 (0.17-24.07)
COV-AID (UNP)	2	17	1	14	1.73 (0.14-21.39)
Rosas et al,^27^ COVACTA, 2021	4	17	4	10	0.46 (0.09-2.50)
PreToVid (UNP)	8	37	12	42	0.69 (0.25-1.93)
Salama et al,^28^ EMPACTA, 2021	13	61	7	33	1.01 (0.36-2.83)
Gordon et al,^32^ REMAP-CAP, 2021	31	166	40	148	0.62 (0.36-1.06)
Rosas et al,^29^ REMDACTA, 2021	41	286	32	159	0.66 (0.40-1.11)
Abani et al,^31^ RECOVERY, 2021	259	711	300	733	0.83 (0.67-1.02)
**Invasive mechanical ventilation**					
COV-AID (UNP)	3	5	2	6	3.00 (0.25-35.34)
CORIMUNO-TOCI-ICU (UNP)	3	6	1	3	2.00 (0.11-35.81)
Veiga et al,^30^ TOCIBRAS, 2021	3	6	2	5	1.50 (0.14-16.54)
ARCHITECTS (UNP)	0	9	1	10	0.33 (0.01-9.26)
HMO-020-0224 (UNP)	4	16	7	11	0.19 (0.04-1.01)
Rosas et al,^27^ COVACTA, 2021	9	26	7	16	0.68 (0.19-2.44)
Gordon et al,^32^ REMAP-CAP, 2021	22	47	33	68	0.93 (0.44-1.97)
Rosas et al,^29^ REMDACTA, 2021	27	58	7	20	1.62 (0.56-4.64)
Abani et al,^31^ RECOVERY, 2021	97	184	127	221	0.83 (0.56-1.22)

Abbreviations: ARCHITECTS, Trial of Tocilizumab for Treatment of Severe COVID-19; BACC, Boston Area COVID-19 Consortium; CORIMUNO-TOCI-1, Cohort Multiple Randomized Controlled Trials Open-label of Immune Modulatory Drugs and Other Treatments in COVID-19 Patients–Tocilizumab Trial; CORIMUNO-TOCI-ICU, Cohort Multiple Randomized Controlled Trials Open-label of Immune Modulatory Drugs and Other Treatments in COVID-19 Patients–Tocilizumab Trial–Intensive Care Unit; COVACTA, A Study to Evaluate the Safety and Efficacy of Tocilizumab in Patients With Severe COVID-19 Pneumonia; COV-AID, Treatment of COVID-19 Patients With Anti-interleukin Drugs; COVIDOSE2-SS-A, Low-Dose Tocilizumab Versus Standard of Care in Hospitalized Patients With COVID-19 (Substudy A); EMPACTA, A Study to Evaluate the Efficacy and Safety of Tocilizumab in Hospitalized Participants With COVID-19 Pneumonia; HMO-020-0224, The Use of Tocilizumab in the Management of Patients Who Have Severe COVID-19 With Suspected Pulmonary Hyperinflammation; ImmCOVA, Immunomodulation-CoV Assessment; PreToVid, Pre-emptive Tocilizumab in Hypoxic COVID-19 Patients; RECOVERY, Randomised Evaluation of COVID-19 Therapy; REMAP-CAP, Randomized, Embedded, Multifactorial Adaptive Platform Trial for Community-Acquired Pneumonia; REMDACTA, A Study to Evaluate the Efficacy and Safety of Remdesivir Plus Tocilizumab Compared With Remdesivir Plus Placebo in Hospitalized Participants With Severe COVID-19 Pneumonia; TOCIBRAS, Tocilizumab in Patients With Moderate to Severe COVID-19; UNP, unpublished data displayed by the World Health Organization Rapid Evidence Appraisal for COVID-19 Therapies Working Group.^1^

### Tocilizumab’s Association With Subgroup Mortality Benefits

**Figure 1.**
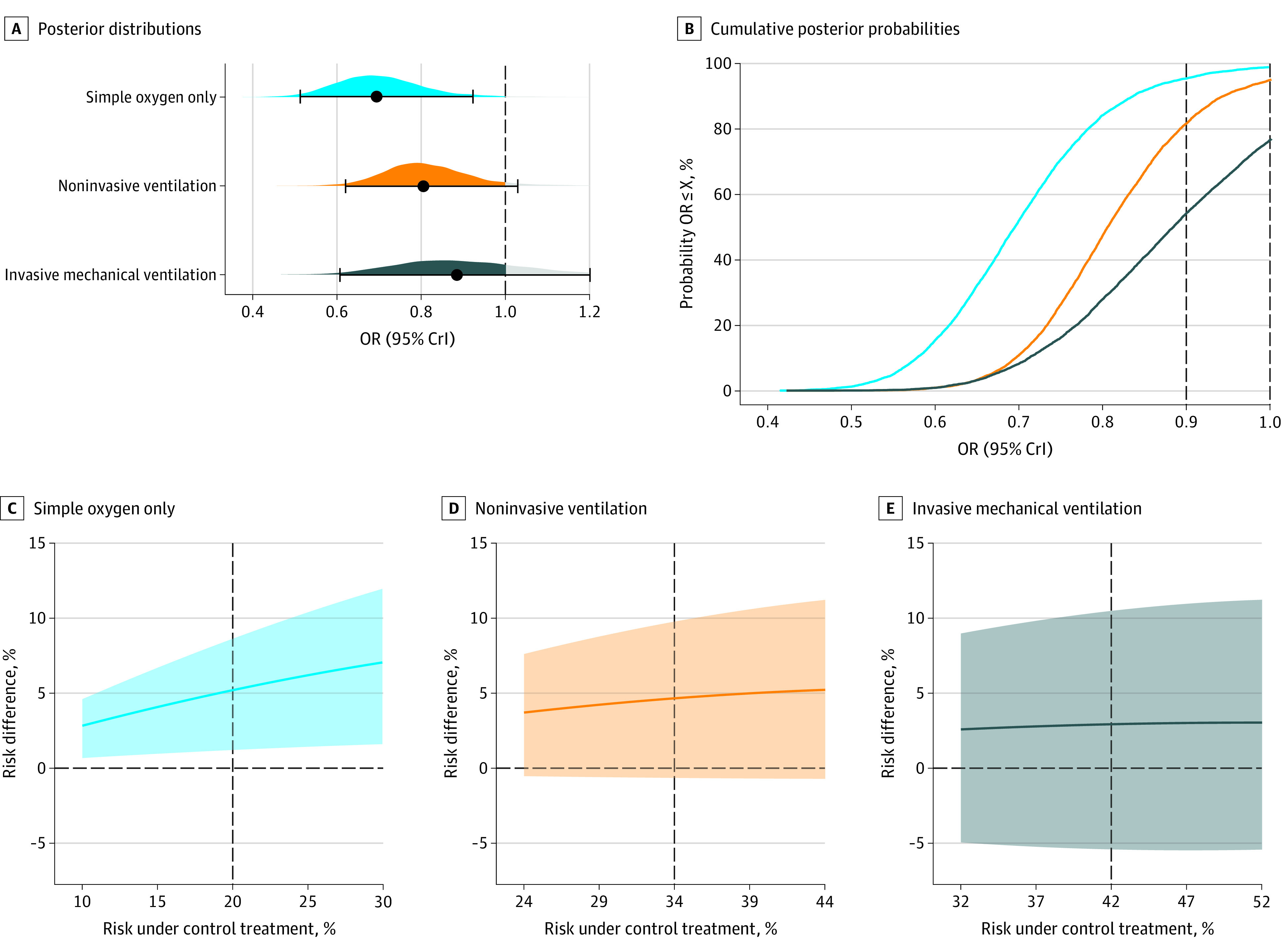
Posterior Distributions and Probabilities Assuming Weakly Informative Priors Results of the meta-analysis assuming weakly informative priors: posterior distributions and probabilities of each subgroup. An odds ratio (OR) lower than 1 indicates reduced mortality owing to tocilizumab in comparison with control treatment. A, Posterior distributions, in which point estimates (black solid-circle data markers) depict the median and interval bars represent the 95% credible (highest-density) intervals (CrIs). B, Cumulative posterior probabilities, which correspond to the probabilities that the OR is lower than or equal to the effect size on the x-axis (X). C through E, Posterior distributions in the risk difference scale (control minus tocilizumab risk) across plausible ranges of mortality risk under control treatment for each subgroup (solid lines represent the median, and shaded areas represent the 95% CrIs). Vertical dashed lines represent the assumed mean risk under control treatment in each subgroup (eMethods in the Supplement). A risk difference greater than 0 indicates reduced mortality owing to tocilizumab in comparison with control treatment. Underlying weakly informative priors are normal, mean (SD) of 0 (0.82) for coefficients and half-normal of 0.5 for the between-study standard deviation.

The separate subgroup marginal posterior distributions, generated with weakly informative priors, are displayed in Figure 1A. In patients receiving simple oxygen only, the median OR was 0.70 (95% CrI, 0.50-0.91), the posterior probability of any benefit association (<1.0 OR) was 98.9% (Figure 1B), and the posterior probability of a meaningful clinical association (OR < 0.9) was 95.5% (Figure 1B). In patients receiving NIV, the median OR was 0.81 (95% CrI, 0.63-1.03), and the posterior probabilities of any and of a meaningful clinical association were 95.5% (Figure 1A) and 82.2% (Figure 1B), respectively. The median OR was 0.89 (95% CrI, 0.61-1.22) among patients receiving IMV, whereas the posterior probabilities of any and of a meaningful clinical association were 75.4% (Figure 1A) and 52.9% (Figure 1B), respectively.

Absolute risk differences (RDs) are more informative for clinical decision-making.^33^ Figure 1C-E shows the posterior distributions of the RDs for the 3 respiratory subgroups as a function of the baseline risk while assuming weakly informative priors. In patients receiving simple oxygen only (Figure 1C), the RD ranged from 2.8% (95% CrI, 0.7-4.6), with baseline risk of 10%, to an RD equal to 7.0% (95% CrI, 1.6-12.0), with baseline risk of 30%. Risk differences for the noninvasive subgroup are shown in Figure 1D. Among patients receiving IMV (Figure 1E), the RD ranged from 2.6% (95% CrI, −4.9 to 9.0), with baseline risk of 32%, to an RD equal to 3.0% (95% CrI, −5.4 to 11.2), with baseline risk of 52%.

Results in the simple oxygen only and NIV subgroups mostly showed associations with probabilities of any and meaningful clinical mortality benefit in the absolute scale (eFigure 1 in the Supplement). The posterior probabilities of clinically meaningful association (risk difference >1%) were greater than 95% and 90% in patients receiving simple oxygen only and NIV, respectively. In contrast, in the IMV subgroup, probabilities of any benefit (>0%) and clinically important associations were 80% and 67%, respectively. The posterior probabilities were mainly stable in each subgroup regardless of the baseline risk.

To assess whether tocilizumab was associated with reduced mortality to a greater extent in a specific subgroup, we calculated the RORs between subgroups (eFigure 2 and eTable 2 in the Supplement).^1^ We found a posterior probability of superiority for the simple oxygen subgroup over the NIV and IMV subgroups of 85.3% and 89.7%, respectively. For the NIV subgroup, we found a posterior probability of superiority of 68.9% over the IMV subgroup.

To test the robustness of these results, we repeated the analyses using 2 different sets of prior distributions (vague and informative). There were no substantial changes in the marginal posterior distributions and probabilities (Table 2 and eTable 2 in the Supplement).

**Table 2.** Posterior Distributions and Probabilities Assuming Distinct Prior Beliefs

Prior/subgroup^a^	OR (95% CrI)	Posterior probability, %		
		OR <0.9	OR <1.0	OR >1.11
Weakly informative				
Simple oxygen only	0.70 (0.50-0.91)	95.5	98.9	0.1
Noninvasive ventilation	0.81 (0.63-1.03)	82.2	95.5	1.2
Invasive mechanical ventilation	0.89 (0.61-1.22)	52.9	75.4	10.1
Vague				
Simple oxygen only	0.68 (0.48-0.91)	95.5	98.4	0.4
Noninvasive ventilation	0.80 (0.60-1.04)	82.3	94.9	1.7
Invasive mechanical ventilation	0.88 (0.60-1.23)	55.9	76.7	9.9
Informative				
Simple oxygen only	0.73 (0.56-0.94)	94.2	98.8	0.2
Noninvasive ventilation	0.83 (0.67-1.03)	77.5	95.0	0.7
Invasive mechanical ventilation	0.91 (0.68-1.23)	46.8	73.1	10.5

Abbreviations: CrI, credible (highest-density) interval; OR, odds ratio.

^a^ Weakly informative priors: mean (SD) normal coefficients of 0 (0.82); between-study standard deviation half-normal, 0.5. Vague priors: mean (SD) normal coefficients of 0 (4); between-study standard deviation half-normal, 4. Informative priors: mean (SD) normal coefficients, 0 (0.35); between-study mean (SD) log-normal, −1.975 (0.67).

### Between-Study Heterogeneity

While assuming weakly informative priors, most of the posterior distribution for the between-study standard deviation (eFigure 3 in the Supplement) was concentrated around low or reasonable levels of heterogeneity (eTable 3 in the Supplement).^6,12^ These probabilities did not notably change on the use of different priors (eTable 3 in the Supplement).

### Posterior Predictive Distributions

**Figure 2.**
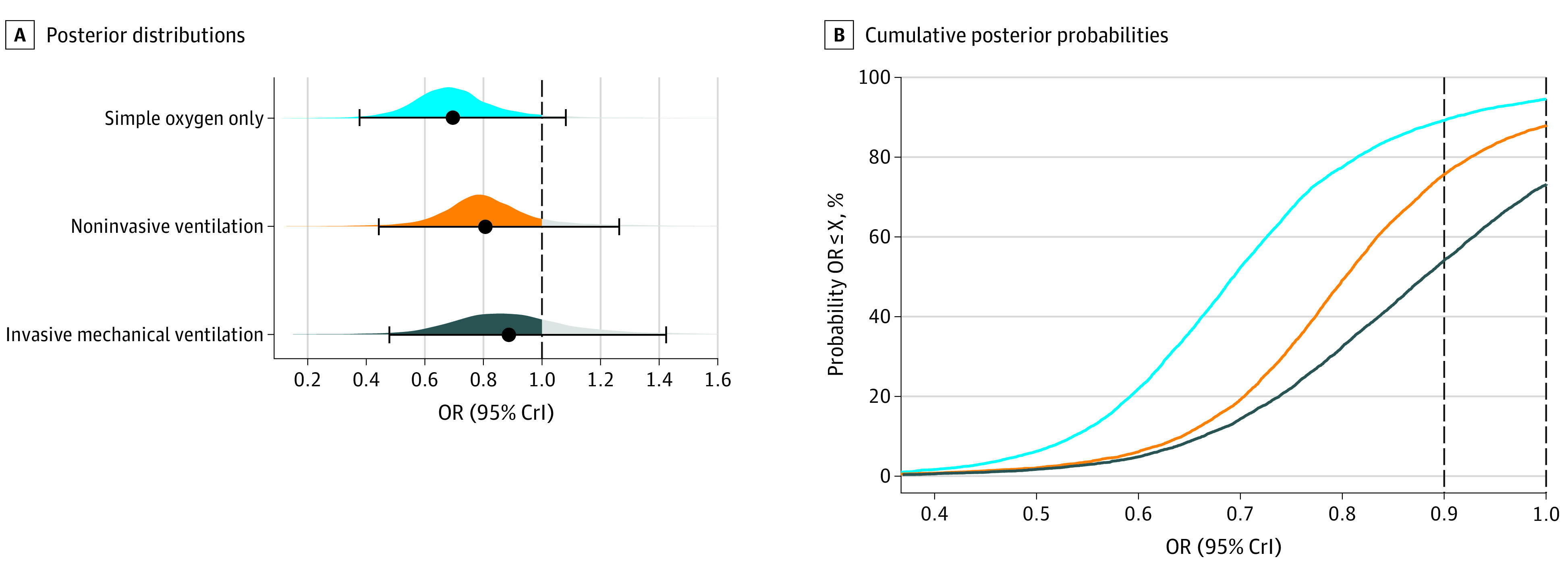
Posterior Predictive Distributions and Probabilities Assuming Weakly Informative Priors Posterior predictive distributions and probabilities of each subgroup. A, Posterior predictive distributions, in which point estimates (black solid-circle data markers) depict the median, and interval bars represent the 95% credible (highest-density) intervals (CrIs). B, Cumulative posterior probabilities, which correspond to the probabilities that future studies (specific to each subgroup) will find a point estimate lower than or equal to the effect size on the x-axis (X). Underlying weakly informative priors are normal, mean (SD) of 0 (0.82) for coefficients and half-normal of 0.5 for the between-study standard deviation. OR indicates odds ratio.

As shown in previous subsections, tocilizumab’s association with mortality benefit in hospitalized patients with COVID-19 receiving IMV is uncertain, as is the probability of clinically meaningful benefits in other respiratory subgroups (Figure 1). Figure 2A and eTable 4 in the Supplement show the predictive distributions for future trial populations for each subgroup while assuming weakly informative priors. The simple oxygen only subgroup showed large benefit (OR, 0.69; 95% CrI, 0.40-1.09) but now acknowledges that the next trial has a small possibility of showing harm (posterior probability of 5.6% for OR >1). In patients receiving NIV, the median OR was 0.80 (95% CrI, 0.46-1.25) along with a posterior probability of 12.4% for harm. The median predictive OR was 0.88 (95% CrI, 0.47-1.40) in the IMV group, and 27.9% of the area under the curve is to the right of 1, representing the probability of potential harm.

In a bayesian framework, one can also calculate the probability that future study populations will find a mean effect size less than a specific threshold (Figure 2B; eTable 4 in the Supplement). Regarding future study populations that exclusively include patients receiving simple oxygen only, there is a 94.2% probability they will find any benefit (<1 OR) and an 88.9% probability for greater benefit (OR <0.9). In contrast, regarding future studies that exclusively include patients receiving IMV, these probabilities were lower (72.1% and 53.3%, respectively).

### Generating RCTs of Patients Receiving IMV

The results depicted in Figure 1A and E and Figure 2A highlight the uncertainty regarding the association of tocilizumab with mortality benefit, especially in patients receiving IMV. One could argue that a possible explanation for this uncertainty is the low total number of deaths (n = 357) and patients (n = 717) in this subgroup (Table 1), yielding a low statistical power to reach definitive conclusions. Therefore, it would be of interest to know the required sample size of a future trial meant to diminish this uncertainty. Bayesian analysis allows one to update posterior distributions with new (real or generated) RCTs of different sample sizes (eTable 5 in the Supplement).

**Figure 3.**
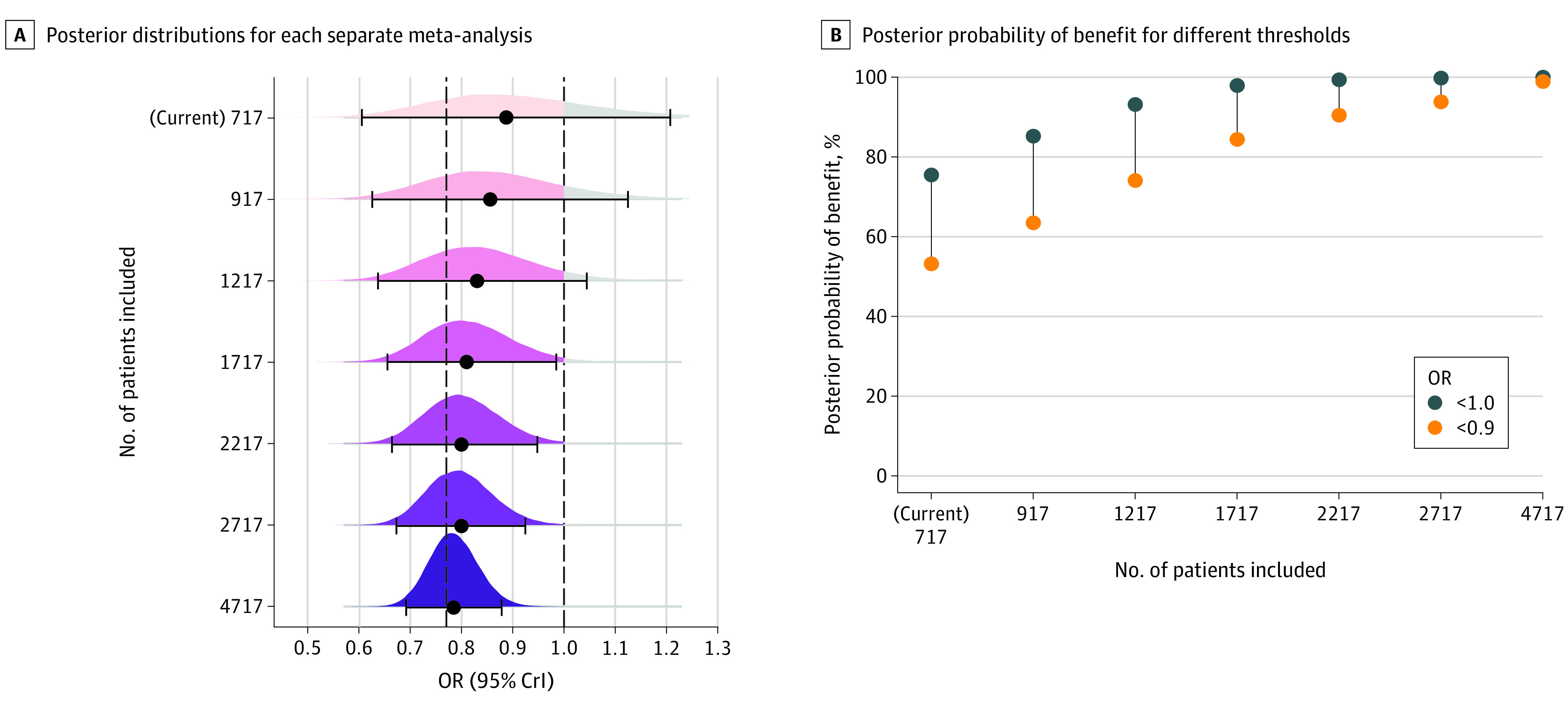
Posterior Distributions and Probabilities From Normal Conjugate Analyses on the Invasive Mechanical Ventilation Subgroup Results for the invasive mechanical ventilation subgroup from meta-analyses using an informative prior based on simulated randomized clinical trials (eTable 5 in the Supplement). “(Current) 717” depicts the results previously shown in Figure 1A and B for this subgroup. A, Posterior distributions for each separate meta-analysis. The y-axis depicts the number of total patients receiving invasive mechanical ventilation included in each respective model (current plus simulated patients). Point estimates (black solid-circle data markers) depict the median, and interval bars represent the 95% credible (highest-density) intervals (CrIs). The vertical dashed line represents a 0.77 odds ratio (OR; World Health Organization Rapid Evidence Appraisal for COVID-19 Therapies Working Group mean result for tocilizumab- and corticosteroid-treated patients).^1^ This value is the mean OR underlying the generated randomized clinical trials (eTable 5 in the Supplement). B, Posterior probability of benefit for different thresholds (OR <1.0 and <0.9). Underlying weakly informative priors are normal, mean (SD) of 0 (1.5) for coefficients and half-normal of 0.5 for the between-study standard deviation.

Figure 3 shows the marginal posterior distributions and probabilities of these analyses. On the addition of more patients, the current evidence (717 patients) is increasingly dominated by new data (Figure 3A; eFigure 4 in the Supplement). For example, current evidence shows that there is only 75.4% probability of any benefit (<1 OR) in patients receiving IMV (Figure 1B and Figure 3B). However, on the addition of a potential future RCT (with an OR = 0.77 as in the WHO REACT meta-analysis on all patients treated with corticosteroids) of 1500 patients, the posterior probability of any benefit becomes greater than 99% (if 2217 patients were included; Figure 3B). Moreover, in a future potential RCT with the same effect size, enrolling 2000 patients (if 2717 patients were included; Figure 3B) will provide a posterior probability of an association with meaningful clinical outcome (<0.9 OR) greater than 95%.

On the other hand, sensitivity analyses combining our current invasive subgroup evidence with a potential future RCT enrolling 1500 patients showing a null effect (OR = 1.0) results in a posterior distribution with only a 20.9% probability of a meaningful clinical association (OR<0.9), suggesting that the futility threshold has likely been reached (eTable 6 and eFigure 5 in the Supplement).

## Discussion

Herein we report our bayesian reanalysis of tocilizumab in COVID-19 respiratory support subgroups. The posterior probabilities of any benefit (OR <1) were notably different between patients on simple oxygen only and IMV (98.9% vs 75.4%). While the point estimates for benefit with tocilizumab were similar across different subgroups, the uncertainties and the probabilities of clinically meaningful association varied substantially, perhaps in part owing to differences in sample size and statistical power. Patients receiving simple oxygen only showed the largest benefit with tocilizumab and the most certainty of being associated with a clinically meaningful benefit, indicated by a greater than 94% probability that the mortality risk difference is greater than 1%. The probabilities of tocilizumab superiority in the simple oxygen subgroup compared with the noninvasive and invasive mechanical subgroups were 85.3% and 89.7%, respectively. Although we found evidence for low or “reasonable” between-study heterogeneity, predictive intervals highlighted that only 72% of studies for patients receiving IMV will demonstrate any benefit. Through bayesian normal conjugate analyses, we also found that a future tocilizumab IMV RCT (with an effect size compatible with what has been observed to date) would require approximately 2000 patients to find an association with a probability of meaningful clinical outcome in this subgroup.

### Comparison With Other Studies

In the WHO REACT Working Group publication,^1^ the authors state, “These differences between subgroups may have arisen due to sampling variation.”^1^^(p515)^ Here, we present a more in-depth analysis and interpretation of these results. First, the association with posterior probabilities of any and of clinically important mortality benefits is strikingly different across subgroups (Figure 1). Second, we estimated the probability that the association of tocilizumab with mortality benefit is different between subgroups (eFigure 2 in the Supplement). Third, we estimated the prediction intervals in each subgroup, reflecting both the uncertainty around the association in each subgroup and the heterogeneity between studies. Prediction intervals showed that one could expect a large proportion of future studies (15%) on patients receiving IMV only to find an association with clinically meaningful harm (ie, OR >1.11; eTable 4 in the Supplement). Fourth, bayesian normal conjugate analysis highlighted that we would require a 2000-patient RCT with a substantial benefit to provide an association with a probability that tocilizumab reduces mortality in patients receiving IMV. Ultimately, we revealed a clear contrast between the strength of the mortality associations for the IMV subgroup and patients with less severe COVID-19, indicating the need for more evidence of tocilizumab’s benefit in critically ill patients.

Our research group has previously reanalyzed the association of tocilizumab with clinical outcomes in different COVID-19 subgroups of patients while focusing on the RECOVERY trial.^34^ However, we did not collect and analyze data limited to patients using corticosteroids in different respiratory subgroups. The lack of this restriction is an important limitation because there is evidence that corticosteroids may synergistically interact with tocilizumab.^1^ Moreover, guidelines suggest that all hospitalized patients with COVID-19 who are receiving supplemental oxygen should receive corticosteroids.^2^ Thus, in this work, we conditioned our results and conclusions to the use of corticosteroids, which limit potential biases if tocilizumab’s association with mortality benefit depends on the use of corticosteroids.

### Limitations

Notwithstanding these strengths, our study also has limitations. First, this reanalysis was not preplanned and should be interpreted as exploratory. Second, we did not have access to patient-level data for any RCTs included in this article. Subgroup analyses that separate patients by a single baseline characteristic are oversimplified and can bring shortcomings.^33^ Lack of patient-level data does not allow analyses using more complex statistical models that incorporate multiple characteristics.^35^ Nevertheless, by limiting these analyses to patients using corticosteroids, we believe concerns about this limitation were mitigated.^1^ Third is the classical argument of bayesian subjectivity owing to the need for priors.^36^ Although we agree with the need for transparency and caution in the choice of priors, the use of weakly informative priors is likely to furnish the most accurate and least biased treatment estimates. Moreover, we also applied different priors yielding similar results, confirming the robustness of the main analysis. Fourth, we limited our focus to a single outcome: 28-day all-cause mortality. Fifth, while the WHO REACT Working Group included other drugs in their meta-analysis,^1^ we limited our reanalysis to tocilizumab because we did not want to introduce further uncertainties about possible drug equivalences. Sixth, we combined within-trial and across-trial interaction estimates to compare subgroups, which could subject our analysis to ecological bias.^37^

## Conclusions

In this bayesian reanalysis of a previous meta-analysis of 15 RCTs, patients with COVID-19 receiving simple oxygen only or noninvasive ventilation and receiving tocilizumab treatment were associated with a probability of a clinically meaningful mortality benefit. In contrast, the beneficial association of this drug was uncertain in patients receiving IMV, and increased harm was not conclusively excluded. Future research should better define whether patients receiving IMV also benefit from tocilizumab.

## Notes

Supplement. **eMethods.** Supplemental Methods
eReferences
**eTable 1.** Demographic Information and Total Number of Patients in Each Subgroup per Study **eFigure 1.** Posterior Probabilities of Benefit in the Risk Difference Scale **eFigure 2.** Posterior Distributions of Relative Difference Between Subgroups **eTable 2.** Posterior Distributions and Probabilities of Relative Difference Between Subgroups While Assuming Distinct Prior Beliefs **eFigure 3.** Posterior Distributions of the Between-Study Standard Deviation While Assuming Distinct Prior Beliefs **eTable 3.** Posterior Distributions and Probabilities of the Between-Study Standard Deviation While Assuming Distinct Prior Beliefs **eTable 4.** Predictive Posterior Distributions and Probabilities While Assuming Weakly Informative Priors **eTable 5.** Description of the Underlying Generated RCTs Used in the Normal Conjugate Analysis (Mean Equal to 0.77) **eFigure 4.** Prior, Data, and Posterior Distributions in the Normal Conjugate Analysis (Data Mean Equal to 0.77) **eTable 6.** Description of the Underlying Generated RCT Used in the Normal Conjugate Analysis (Mean Equal to 1.00) **eFigure 5.** Prior, Data, and Posterior Distributions in the Normal Conjugate Analysis (Data Mean Equal to 1.00)
