# Errors in Figures

**DOI:** 10.1001/jamanetworkopen.2022.5937

**Published:** 2022-03-22

In the Original Investigation titled “Mortality Rates Among Hospitalized Patients With COVID-19 Infection Treated With Tocilizumab and Corticosteroids: A Bayesian Reanalysis of a Previous Meta-analysis,”^1^ published February 28, 2022, errors occurred in 2 figures. In Figures 1B and 2B, the x-axis label was incorrectly published as “OR (95% CI)”; that label should have appeared as “OR.” This article has been corrected.^1^
