# Supplementary material for: Mortality Rates Among Hospitalized Patients With COVID-19 Infection Treated With Tocilizumab and Corticosteroids: A Bayesian Reanalysis of a Previous Meta-analysis
Source: JAMA Netw Open. 2022 Feb 28;5(2):e220548. doi: 10.1001/jamanetworkopen.2022.0548 (PMC8886529; doi:10.1001/jamanetworkopen.2022.0548)
Supplement: Supplement. — eMethods. Supplemental Methods eReferences eTable 1. Demographic Information and Total Number of Patients in Each Subgroup per Study eFigure 1. Posterior Probabilities of Benefit in the Risk Difference Scale eFigure 2. Posterior Distributions of Relative Difference Between Subgroups eTable 2. Posterior Distributions and Probabilities of Relative Difference Between Subgroups While Assuming Distinct Prior Beliefs eFigure 3. Posterior Distributions of the Between-Study Standard Deviation While Assuming Distinct Prior Beliefs eTable 3. Posterior Distributions and Probabilities of the Between-Study Standard Deviation While Assuming Distinct Prior Beliefs eTable 4. Predictive Posterior Distributions and Probabilities While Assuming Weakly Informative Priors eTable 5. Description of the Underlying Generated RCTs Used in the Normal Conjugate Analysis (Mean Equal to 0.77) eFigure 4. Prior, Data, and Posterior Distributions in the Normal Conjugate Analysis (Data Mean Equal to 0.77) eTable 6. Description of the Underlying Generated RCT Used in the Normal Conjugate Analysis (Mean Equal to 1.00) eFigure 5. Prior, Data, and Posterior Distributions in the Normal Conjugate Analysis (Data Mean Equal to 1.00) [file jamanetwopen-e220548-s001.pdf]

Supplementary Online Content

Albuquerque AM, Tramujas L, Sewanan LR, Williams DR, Brophy JM. Mortality rates among hospitalized patients with COVID-19 infection treated with tocilizumab and corticosteroids: a bayesian reanalysis of a previous meta-analysis. *JAMA Netw Open*. 2022;5(2):e220548. doi:10.1001/jamanetworkopen.2022.0548

eMethods. Supplemental Methods

eReferences

eTable 1. Demographic Information and Total Number of Patients in Each Subgroup per Study

eFigure 1. Posterior Probabilities of Benefit in the Risk Difference Scale

eFigure 2. Posterior Distributions of Relative Difference Between Subgroups

eTable 2. Posterior Distributions and Probabilities of Relative Difference Between Subgroups While Assuming Distinct Prior Beliefs

eFigure 3. Posterior Distributions of the Between-Study Standard Deviation While Assuming Distinct Prior Beliefs

eTable 3. Posterior Distributions and Probabilities of the Between-Study Standard Deviation While Assuming Distinct Prior Beliefs

eTable 4. Predictive Posterior Distributions and Probabilities While Assuming Weakly Informative Priors

eTable 5. Description of the Underlying Generated RCTs Used in the Normal Conjugate Analysis (Mean Equal to 0.77)

eFigure 4. Prior, Data, and Posterior Distributions in the Normal Conjugate Analysis (Data Mean Equal to 0.77)

eTable 6. Description of the Underlying Generated RCT Used in the Normal Conjugate Analysis (Mean Equal to 1.00)

eFigure 5. Prior, Data, and Posterior Distributions in the Normal Conjugate Analysis (Data Mean Equal to 1.00)

This supplementary material has been provided by the authors to give readers additional information about their work.

Supplemental Content

Table of Contents

eMethods 3
Bayesian meta-analysis 3
Weakly informative priors 3
Alternative priors 5
Deriving risk difference from odds ratio 7
Predictive analysis to confirm tocilizumab's association with mortality benefit 8
Prior 8
Likelihood 8
References 11
eFigures and eTables 12
eTable 1. Demographic Information and Total Number of Patients in Each Subgroup per Study 12
eFigure 1. Posterior Probabilities of Benefit in the Risk Difference Scale 13
eFigure 2. Posterior Distributions of Relative Difference Between Subgroups 13
eTable 2. Posterior Distributions and Probabilities of Relative Difference Between Subgroups While Assuming Distinct Prior Beliefs 14
eFigure 3. Posterior Distributions of the Between-Study Standard Deviation While Assuming Distinct Prior Beliefs 15
eTable 3. Posterior Distributions and Probabilities of the Between-Study Standard Deviation While Assuming Distinct Prior Beliefs 15
eTable 4. Predictive Posterior Distributions and Probabilities While Assuming Weakly Informative Priors 16
eTable 5. Description of the Underlying Generated RCTs Used in the Normal Conjugate Analysis (Mean Equal to 0.77) 16
eFigure 4. Prior, Data, and Posterior Distributions in the Normal Conjugate Analysis (Data Mean Equal to 0.77) 17
eTable 6. Description of the Underlying Generated RCT Used in the Normal Conjugate Analysis (Mean Equal to 1.00) 18
eFigure 5. Prior, Data, and Posterior Distributions in the Normal Conjugate Analysis (Data Mean Equal to 1.00) 19

eMethods. Supplemental Methods

Bayesian meta-analysis

Our random-effect model is defined as:

$$\begin{aligned} y_i &\sim Normal(\theta_i, \sigma_i^2) \\ \theta_i &\sim Normal(\mu, \tau^2) \\ \mu &= \alpha_{subgroup[k]} \end{aligned}$$

where y_i is the observed mean log odds ratio of tocilizumab versus control and σ_i^2 is the known sampling variance in study i. Because this is a random-effect model, each study i has its own distribution, where θ_i represents its mean effect. All θ_is are drawn from normal distribution where the mean effect is μ and the variance τ^2, which represents the between-study heterogeneity. μ is predicted by a no-intercept linear regression, where each subgroup k (simple oxygen only; noninvasive ventilation; invasive mechanical ventilation) has its own parameter α, representing the effect of each respective subgroup.

In this case, we can assess tocilizumab's effect in each subgroup while assuming a common between-study heterogeneity:

- Simple oxygen only = α_{SOO}
- Noninvasive ventilation = α_{NIV}
- Invasive mechanical ventilation = α_{IMV}

Weakly informative priors

Because we applied the Bayesian framework, we assigned a prior distribution to each parameter. In our main model, we implemented priors that cover plausible values for all parameters, assigning limited density to impossible values, and thus employed little influence in the results (hereafter, known as weakly informative priors).[1,2] These are our weakly informative priors:

$$\begin{aligned} \alpha &\sim \text{Normal}(0, 0.82^2) \\ \tau &\sim \text{Half-Normal}(0.5^2) \end{aligned}$$

Now, we will explain the rationale underlying these distributions.

We find highly unlikely that a pharmacological treatment, such as tocilizumab, will yield a 80% odds reduction in 28-days all-cause mortality regardless of the subgroup of patients, as suggested by empirical evidence.[3] Thus, for α, we set a prior distribution of $Normal(0, 0.82)$ in the log odds ratio scale.

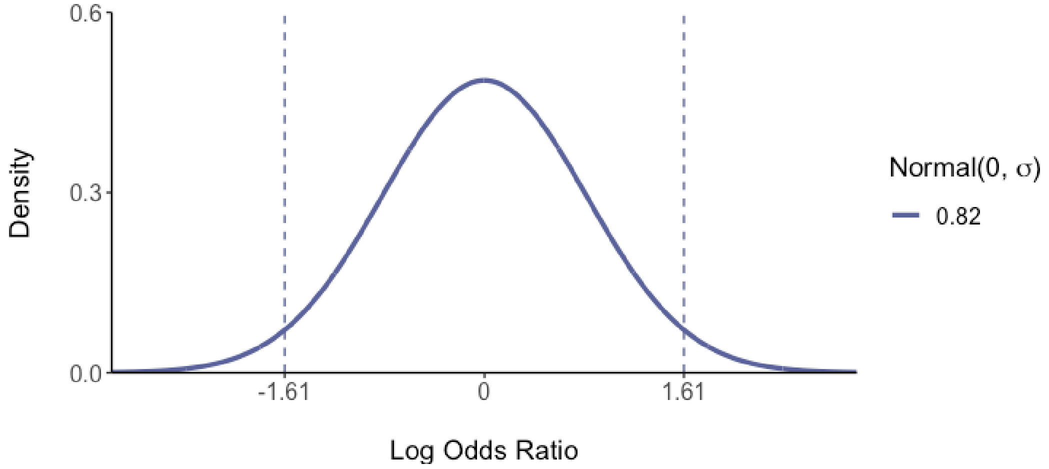

To facilitate the understanding of these distributions, here are the corresponding 95% quantile intervals in the linear scale.

Log scale		Linear scale	
Mean	SD	Mean	95% CI
0	0.82	1	[0.2, 5]

Another way to assess the plausibility of the priors is to perform a prior predictive check[4], which can be visualized below:

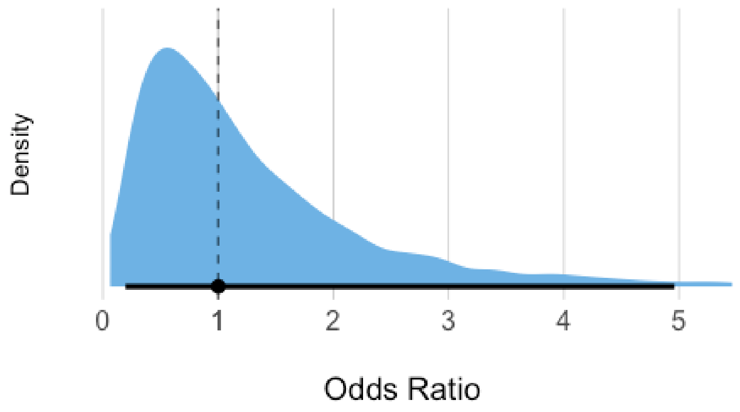

Point estimate depicts the median and interval bar depicts the 95% credible (quantile) interval.

As expected, the distribution approximately ranges from 0.2 to 5.0.

Lastly, we will now discuss the weakly informative prior distribution for τ. Because we wanted to perform unconditional inferences beyond the included studies, we fitted a random-effect meta-analysis. In this model, one assumes there is within-study heterogeneity (represented by σ_i^2, the known sampling variance in study i) and the between-study heterogeneity (represented by τ).

Although the definition of small or large between-study heterogeneity is arbitrary, previous work suggests cutoff values ("reasonable" heterogeneity between 0.1 and 0.5, "fairly high" between 0.5 and 1.0, and "fairly extreme" for values larger than 1.0 log odds ratio).[2,5] We added a category for low heterogeneity (between 0 and 0.1).

The Half-Normal(0.5) distribution yields plausible probabilities in each of these ranges:

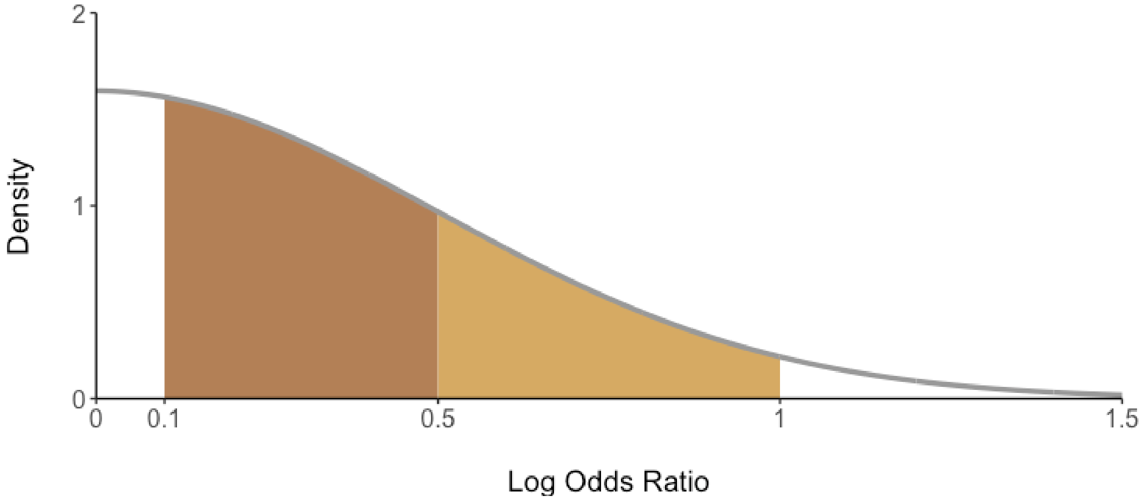

Here are the corresponding probabilities within each of the heterogeneity ranges:

Heterogeneity Range			
Low	Reasonable	Fairly High	Fairly Extreme
16%	52%	27%	5%

Alternative priors

To check whether the choice of weakly informative priors meaningfully impacted our results or our conclusions, we also fitted models using vague or informative priors.

Vague priors:

$$\begin{aligned} \alpha &\sim \text{Normal}(0, 4^2) \\ \tau &\sim \text{Half-Normal}(4^2) \end{aligned}$$

Informative priors:[6]

$$\begin{aligned} \alpha &\sim \text{Normal}(0, 0.35^2) \\ \tau &\sim \text{Log-Normal}(-1.975, 0.67^2) \end{aligned}$$

Here are graphical representations of these normal distributions (along with the weakly informative mentioned before):

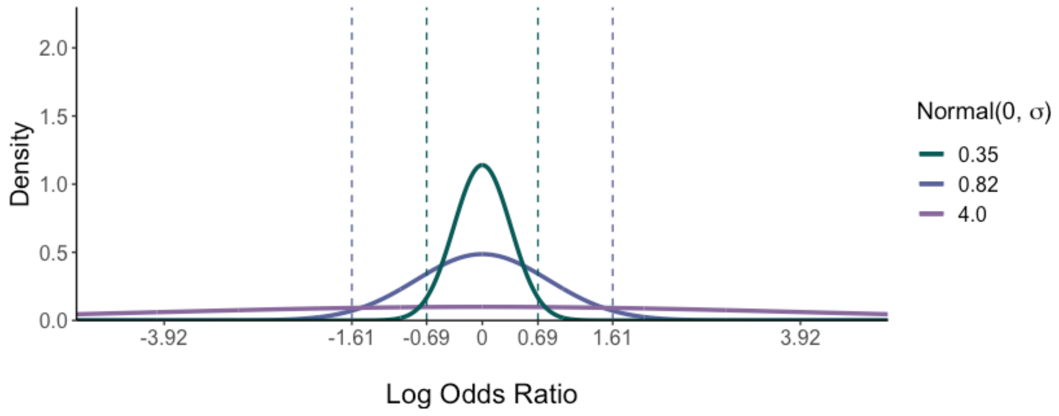

Log scale		Linear scale	
Mean	SD	Mean	95% CI
0	0.35	1	[0.5, 2]
0	0.82	1	[0.2, 5]
0	4.00	1	[0, 2540.2]

Here are graphical representations of distributions for the between-study standard deviation (τ) (along with the weakly informative mentioned before):

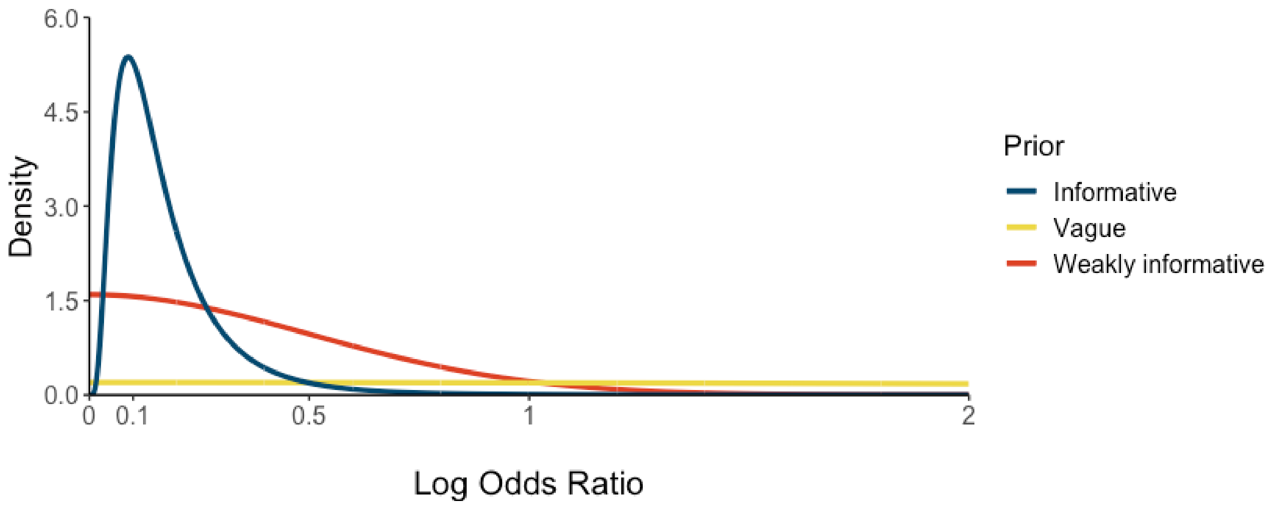

		Heterogeneity Range			
Prior	Distribution	Low	Reasonable	Fairly High	Fairly Extreme
Weakly informative	Half-Normal(0.5)	16%	52%	27%	5%
Vague	Half-Normal(4.0)	2%	8%	10%	80%
Informative	Log-Normal(-1.975, 0.67)	31%	66%	3%	0%

Deriving risk difference from odds ratio

We used the odds ratio as our primary estimand.[11,14]

We derived the risk in the tocilizumab group using the following formula:[11]

$$R_T = \frac{R_C OR}{R_C(OR - 1) + 1}$$

where R_T is the mortality risk in the tocilizumab group, R_C is the mortality risk in the control group and OR is the odds ratio.

We then calculated the risk difference (RD) with the following formula:[11]

$$RD = R_T - R_C$$

We assumed different mortality risks in each subgroup. For the simple oxygen only and noninvasive ventilation subgroups, we used the average mortality risk in each subgroup based on the data included in this reanalysis. In contrast, regarding the invasive mechanical ventilation (IVM) subgroup, we found a striking discrepancy between the control mortality risk in the data included in this reanalysis (52%) in comparison to another previously published meta-analysis (34% in patients on IVM and using corticosteroids).[10] Thus, we have decided to use 43% (arithmetic mean between 34 and 52) as our reference risk in the IVM subgroup. Recognizing the potential variability of the subgroup baseline risks, we estimated the risk differences with twenty different plausible baseline risks for each subgroup (spanning +- 10% change from the reference risks mentioned above).

Subgroup	Control Risk
Simple oxygen only	20 +- 10%
Noninvasive ventilation	34 +- 10%
Invasive mechanical ventilation	42 +- 10%

Predictive analysis to confirm tocilizumab's association with mortality benefit

In brief, we will update our current evidence (as modeled in our main model) with generated randomized clinical trials (RCTs) of different sample sizes comparing tocilizumab to control on patients on invasive mechanical ventilation.

We will use the estimated marginal posterior mean and standard error on this subgroup to create a prior distribution. Then, we will use normal conjugate analyses to update this prior with new data (likelihood) and form updated posterior distributions.

Prior

As described before, we fitted a Bayesian meta-analysis model, from which we estimated marginal posterior distributions on different subgroups. Once again using a Bayesian approach, we updated our current belief, as expressed by the results of our current Bayesian meta-analysis, and which has now become our new prior, with the results of these new generated RCTs to arrive at revised posterior distributions.

Of note, the only subgroup of interest now is the invasive mechanical ventilation:

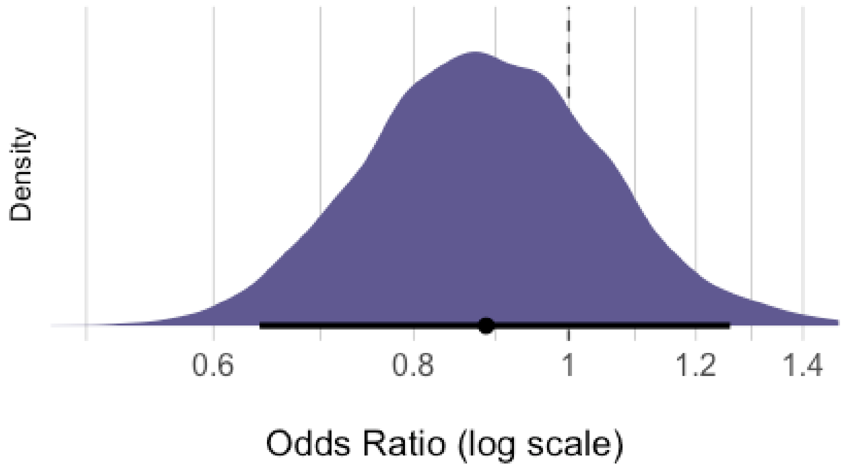

Marginal posterior distribution of the invasive mechanical ventilation subgroup (also depicted in Figure 1A). The interval bar depicts the mean and 95% credible (quantile) interval.

In the linear scale, the mean of this marginal posterior distribution is 0.89. Because we will use normal conjugate analysis, it is of greater interest to evaluate this distribution on the log scale, which is approximately normally distributed. In this case, the mean is -0.12 and the standard error is 0.17.

Thus, in the following normal conjugate analyses, we will use the following distribution as our prior:

$$\text{Normal}(-0.12, 0.17^2)$$

Likelihood

We created six different RCTs and update the prior distribution mentioned above six separate times.

Assuming the prior is normally distributed

$$N(\theta, \sigma^2)$$

and so is the data (likelihood).

$$N(\hat{\theta}, \hat{\sigma}^2)$$

The mean and variance of the posterior distributions can be estimated by the following formulas:[5]

- Mean

$$\frac{\frac{\theta}{\sigma^2} + \frac{\hat{\theta}}{\hat{\sigma}^2}}{\frac{1}{\sigma^2} + \frac{1}{\hat{\sigma}^2}}$$

- Variance

$$\frac{1}{\frac{1}{\sigma^2} + \frac{1}{\hat{\sigma}^2}}$$

In summary, we can update a normally distributed prior distribution (shown in the Figure above) with normally distributed data to generate a normally distributed posterior distribution. Based on the posterior's mean and variance, we generated 100,000 random samples (seed number of 123).

Now, we must decide the mean and standard deviation of the likelihood. All RCTs will have a mean of -0.26 (log scale). This value is the equal to 0.77 in the linear scale, which was chosen based on WHO's meta-analysis (page 14 in their Supplement 2).[7] This is the mean odds ratio of tocilizumab vs. control in patients using corticosteroids (overall results). We decided to use this value to reflect a skeptical view to heterogeneity of treatment effect across subgroups,[8] and thus the "real" effect in this subgroup would be equal to the largest body of evidence available for tocilizumab in all hospitalized COVID-19 patients on corticosteroids.

Given that all six generated RCTs were set to find the same effect size, the only difference between them was the total number of included patients: 200, 500, 1000, 1500, 2000, or 4000. To calculate the standard deviation of each corresponding prior based on the number of total patients included, one must also assume the proportion of patients included in each treatment arm and the mortality risk in the control arm:

1. We assumed equal allocation in both treatment arms
2. Adapting from the suggestions in the GRADE guidelines,[9(p12)] we found a striking discrepancy between the control mortality risk in the data included in this reanalysis (52%) in comparison to another previously published meta-analysis(34% in patients on IVM and using corticosteroids).[10] Thus, we have decided to use 43% (arithmetic mean between 34 and 52) as our reference risk in the IVM subgroup
3. The mortality risk in the tocilizumab was calculated using the following formula:[11]

$$R_T = \frac{R_C OR}{R_C(OR - 1) + 1}$$

where R_T is the mortality risk in the tocilizumab group, R_C is the mortality risk in the control group and OR is the odds ratio mentioned above. Thus, the tocilizumab risk is equal to 37%.

In summary, we are generating RCTs with mean OR equal to 0.77, control risk mortality of 43%, and tocilizumab risk of 37%.

Based on these values, we can estimate the standard deviation (SD) with the following formula:[12]

$$SD = \sqrt{\frac{1}{a + \frac{1}{2}} + \frac{1}{b + \frac{1}{2}} + \frac{1}{c + \frac{1}{2}} + \frac{1}{d + \frac{1}{2}}}$$

where a, b, c and d are number of events and follow this 2x2 table:

Event	Tocilizumab	Control
Death	a	c
No death	b	d

As previous work has shown,[13] we can estimate these values as:

- $a = R_T SS_T$
- $b = SS_T - R_T SS_T$
- $c = R_C SS_C$
- $d = SS_C - R_C SS_C$

where SS_T and SS_C are the sample sizes in the tocilizumab and control arms, respectively. As mentioned above, we assume equal allocation in both treatment arms, thus $SS_T = SS_C$.

Finally, the SDs based on the 6 different sample sizes mentioned above are:

Sample size in each treatment arm	Total sample size	SD
100	200	0.29
250	500	0.18
500	1,000	0.13
750	1,500	0.11
1,000	2,000	0.09
2,000	4,000	0.06

eReferences

1. van de Schoot R, Veen D, Smeets L, Winter SD, Depaoli S. A Tutorial on Using The Wambs Checklist to Avoid The Misuse of Bayesian Statistics. In: van de Schoot R, Miočević M, eds. *Small Sample Size Solutions*. 1st ed. Routledge; 2020:30-49. doi:10.4324/9780429273872-4

2. Röver C, Bender R, Dias S, et al. On weakly informative prior distributions for the heterogeneity parameter in Bayesian random-effects meta-analysis. *Res Synth Methods*. 2021;12(4):448-474. doi:10.1002/jrsm.1475

3. Pedroza C, Han W, Thanh Truong VT, Green C, Tyson JE. Performance of informative priors skeptical of large treatment effects in clinical trials: A simulation study. *Stat Methods Med Res*. 2018;27(1):79-96. doi:10.1177/0962280215620828

4. Gabry J, Simpson D, Vehtari A, Betancourt M, Gelman A. Visualization in Bayesian workflow. *J R Stat Soc Ser A Stat Soc*. 2019;182(2):389-402. doi:10.1111/rssa.12378

5. Spiegelhalter DJ, Abrams KR, Myles JP. *Bayesian Approaches to Clinical Trials and Health Care Evaluation*. Wiley; 2004.

6. Turner RM, Jackson D, Wei Y, Thompson SG, Higgins JPT. Predictive distributions for between-study heterogeneity and simple methods for their application in Bayesian meta-analysis. *Stat Med*. 2015;34(6):984-998. doi:10.1002/sim.6381

7. The WHO Rapid Evidence Appraisal for COVID-19 Therapies (REACT) Working Group, Domingo P, Mur I, et al. Association Between Administration of IL-6 Antagonists and Mortality Among Patients Hospitalized for COVID-19: A Meta-analysis. *JAMA*. Published online July 6, 2021. doi:10.1001/jama.2021.11330

8. Kent DM, Steyerberg E, Klaveren D van. Personalized evidence based medicine: predictive approaches to heterogeneous treatment effects. *BMJ*. 2018;363:k4245. doi:10.1136/bmj.k4245

9. Guyatt GH, Oxman AD, Santesso N, et al. GRADE guidelines: 12. Preparing Summary of Findings tables—binary outcomes. *J Clin Epidemiol*. 2013;66(2). doi:10.1016/j.jclinepi.2012.01.012

10. The WHO Rapid Evidence Appraisal for COVID-19 Therapies (REACT) Working Group, Sterne JAC, Murthy S, et al. Association Between Administration of Systemic Corticosteroids and Mortality Among Critically Ill Patients With COVID-19: A Meta-analysis. *JAMA*. 2020;324(13):1330. doi:10.1001/jama.2020.17023

11. Doi SA, Furuya-Kanamori L, Xu C, Lin L, Chivese T, Thalib L. Questionable utility of the relative risk in clinical research: a call for change to practice. *J Clin Epidemiol*. 2020;0(0). doi:10.1016/j.jclinepi.2020.08.019

12. Wijeysundera DN, Austin PC, Hux JE, Beattie WS, Laupacis A. Bayesian statistical inference enhances the interpretation of contemporary randomized controlled trials. *J Clin Epidemiol*. 2009;62(1):13-21.e5. doi:10.1016/j.jclinepi.2008.07.006

13. Higgins JP, Spiegelhalter DJ. Being sceptical about meta-analyses: a Bayesian perspective on magnesium trials in myocardial infarction. *Int J Epidemiol*. 2002;31(1):96-104. doi:10.1093/ije/31.1.96

14. Doi SA, Furuya-Kanamori L, Xu C, et al. The OR is “portable” but not the RR: Time to do away with the log link in binomial regression. *J Clin Epidemiol*. Published online August 2021:S0895435621002419. doi:10.1016/j.jclinepi.2021.08.003

eFigures and eTables

eTable 1. Demographic Information and Total Number of Patients in Each Subgroup per Study

Study	Location	Age[1]	Male, %[1]	Number of Patients in Each Subgroup			Sum
				Simple oxygen only	Noninvasive ventilation	Invasive mechanical ventilation	
ARCHITECTS	USA	61.5	56.80	0	0	19	19
BACC-Bay	USA	59.0	57.50	4	0	0	4
CORIMUNO-TOCI-1	France	63.7	68.00	22	0	0	22
CORIMUNO-TOCI-ICU	France	64.3	72.00	0	3	9	12
COV-AID	Belgium	62.9	75.70	46	31	11	88
COVACTA	Europe and North America	62.3	70.00	23	27	42	92
COVIDOSE2-SS-A	USA	65.0	69.30	7	0	0	7
EMPACTA	North and Latin America; Kenya	56.5	58.60	200	94	0	294
HMO-020-0224	Israel	63.8	65.90	0	19	27	46
ImmCOVA	Sweden	63.0	76.00	0	29	0	29
PreToVid	Netherlands	66.5	67.00	234	79	0	313
RECOVERY	UK	63.9	67.50	1531	1444	405	3380
REMAP-CAP	Europe and Oceania; Saudi Arabia	61.0	71.90	0	314	115	429
REMDACTA	Spain, USA, Brazil, Russian Federation	60.0	64.05	16	445	78	539
TOCIBRAS	Brazil	56.3	67.70	34	20	11	65
Of note, age and male proportion were extracted from the WHO REACT Working Group meta-analysis. They regard the whole sample of patients included in the trial, and not only on patients treated with corticosteroids. Thus, cautious interpretation of these characteristics is warranted.							
[1] Mean value of both treatment arms							

eFigure 1. Posterior Probabilities of Benefit in the Risk Difference Scale

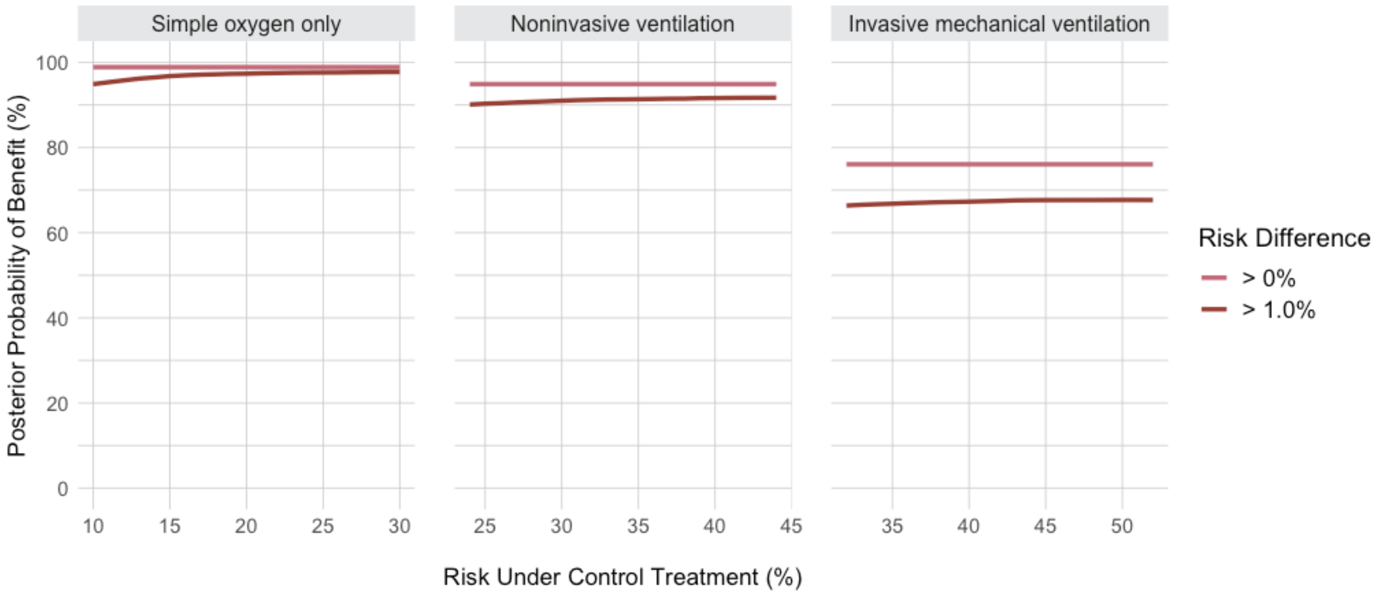

Posterior probabilities of benefit per subgroup in the risk difference scale assuming weakly informative priors. Each line represents the posterior probability of benefit for a specific cutoff, such as risk difference greater than 0% or 1%, across plausible ranges of mortality risk under control treatment. Underlying weakly informative priors are N(0, 0.82) for the coefficients, and HN(0.5) for the between-study standard deviation. N(mu, sigma) = Normal(mean, standard deviation); HN(sigma) = Half-Normal(standard deviation)

eFigure 2. Posterior Distributions of Relative Difference Between Subgroups

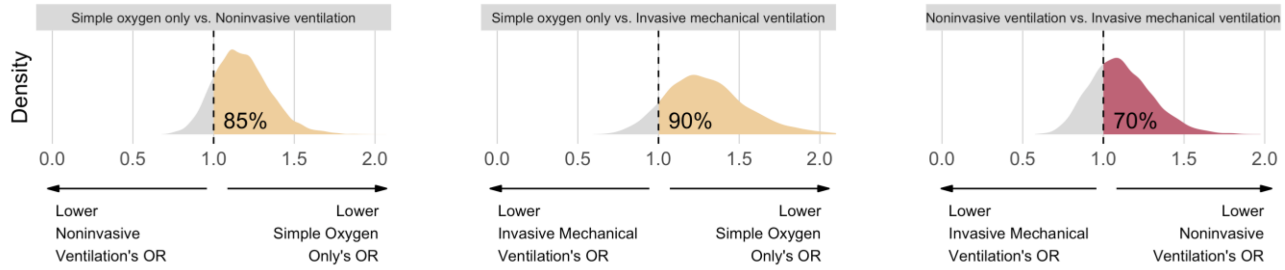

Posterior distributions for comparisons of effect sizes between subgroups while assuming weakly informative priors. Each distribution represents the ratio of odds ratios of two subgroups. On top of each distribution, there is a percentage representing the posterior probability of a ratio of odds ratios greater than 1.0 (eTable 2). Arrows on the bottom represent - in that comparison - which subgroup benefited to a greater extent tocilizumab's effect on mortality reduction (lower odds ratio [OR]). For example, according to our model, there was an 85% probability that tocilizumab reduces mortality to a greater extent in the simple oxygen subgroup in comparison to noninvasive ventilation. Underlying weakly informative priors are N(0, 0.82) for each coefficient, and HN(0.5) for the between-study standard deviation. N(mu, sigma) = Normal(mean, standard deviation); HN(sigma) = Half-Normal(standard deviation).

eTable 2. Posterior Distributions and Probabilities of Relative Difference Between Subgroups While Assuming Distinct Prior Beliefs

Priors[a] / Comparisons	Ratio of Odds Ratios [95% CI]	Pr(> 1.00), %	Pr(> 1.25), %
**Weakly informative[a]**			
Simple oxygen only vs. Noninvasive ventilation	1.17 [0.85, 1.52]	85.3	32.3
Simple oxygen only vs. Invasive mechanical ventilation	1.28 [0.84, 1.84]	89.7	54.4
Noninvasive ventilation vs. Invasive mechanical ventilation	1.10 [0.75, 1.54]	68.9	23.5
**Vague[a]**			
Simple oxygen only vs. Noninvasive ventilation	1.17 [0.84, 1.53]	85.2	33.1
Simple oxygen only vs. Invasive mechanical ventilation	1.29 [0.84, 1.82]	90.0	55.7
Noninvasive ventilation vs. Invasive mechanical ventilation	1.10 [0.72, 1.50]	69.7	23.5
**Informative[a]**			
Simple oxygen only vs. Noninvasive ventilation	1.14 [0.86, 1.49]	82.3	25.9
Simple oxygen only vs. Invasive mechanical ventilation	1.25 [0.86, 1.78]	88.9	49.9
Noninvasive ventilation vs. Invasive mechanical ventilation	1.10 [0.75, 1.47]	70.8	21.6

[a] Weakly informative priors: Coefficients N(0, 0.82); Between-study standard deviation HN(0.5) / Vague priors: Coefficients N(0, 4); Between-study standard deviation HN(4) / Informative priors: Coefficients N(0, 0.35); Between-study standard deviation LN(-1.975, 0.67). N(mu, sigma) = Normal(mean, standard deviation); HN(sigma) = Half-Normal(standard deviation); LN(mu, sigma) = Log-Normal(mean, standard deviation)

Abbreviations: CI, Credible interval; Pr, Posterior probability above specific value

eFigure 3. Posterior Distributions of the Between-Study Standard Deviation While Assuming Distinct Prior Beliefs

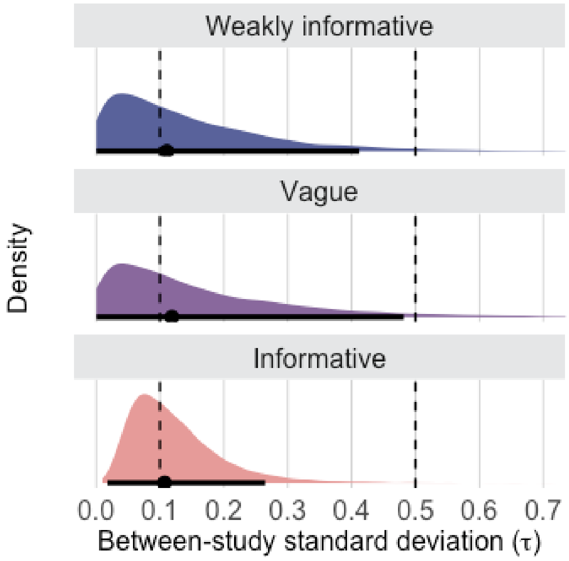

Posterior distributions (log scale) of the between study standard deviation (tau) upon different underlying prior distributions (weakly informative, vague or informative). Tau is a proxy for the between-study heterogeneity in random-effect meta-analyses. Weakly informative priors: Coefficients N(0, 0.82); Between-study standard deviation HN(0.5) / Vague priors: Coefficients N(0, 4); Between-study standard deviation HN(4) / Informative priors: Coefficients N(0, 0.35); Between-study standard deviation LN(-1.975, 0.67). N(mu, sigma) = Normal(mean, standard deviation); HN(sigma) = Half-Normal(standard deviation); LN(mu, sigma) = Log-Normal(mean, standard deviation).

eTable 3. Posterior Distributions and Probabilities of the Between-Study Standard Deviation While Assuming Distinct Prior Beliefs

Underlying Prior	Median [95% CI]	Posterior Probability within Heterogeneity Ranges, %		
		Low	Reasonable	Fairly High
Weakly informative	0.11 [0, 0.41]	46.2	51.4	2.4
Vague	0.12 [0, 0.49]	43.4	52.1	4.3
Informative	0.1 [0.02, 0.26]	45.3	54.6	0.1

Abbreviations: CI, Compatibility (highest density) interval

Although the definition of small or large between-study heterogeneity is arbitrary, previous work suggests cutoff values: “reasonable” heterogeneity between 0.1 and 0.5, “fairly high” between 0.5 and 1.0, and “fairly extreme” for values larger than 1.0 log odds ratio for the between-study standard deviation (tau).[2,5] We added a category for low heterogeneity (between 0 and 0.1). Thus, in this table, we present the posterior probabilities within each one these ranges. Because we fitted three different models using different prior distributions (weakly informative, vague, and informative), here we depict tau posterior distributions/probabilities regarding each model.

Weakly informative priors: Coefficients N(0, 0.82); Between-study standard deviation HN(0.5) / Vague priors: Coefficients N(0, 4); Between-study standard deviation HN(4) / Informative priors: Coefficients N(0, 0.35); Between-study standard deviation LN(-1.975, 0.67). N(mu, sigma) = Normal(mean, standard deviation); HN(sigma) = Half-Normal(standard deviation); LN(mu, sigma) = Log-Normal(mean, standard deviation)

eTable 4. Predictive Posterior Distributions and Probabilities While Assuming Weakly Informative Priors

Subgroup	Odds Ratio [95% CI]	Pr(< 0.9), %	Pr(< 1.00), %	Pr(> 1.11), %
Simple oxygen only	0.69 [0.40, 1.09]	89	94	3
Noninvasive ventilation	0.80 [0.46, 1.25]	75	88	7
Invasive mechanical ventilation	0.88 [0.47, 1.40]	53	72	15

Abbreviations: CI, Compatibility (highest density) interval; Pr, Posterior probability below or above specific odds ratio
Weakly informative priors: Coefficients N(0, 0.82); Between-study standard deviation HN(0.5)

eTable 5. Description of the Underlying Generated RCTs Used in the Normal Conjugate Analysis (Mean Equal to 0.77)

Model	Generated RCT Characteristics				Number of Patients Included[a]
	Evidence Equivalence	Mean [95% CI]	Pr(OR < 1.0), %	Pr(OR < 0.9), %	
Main Model	-	1 [0.2, 4.99]	50	45	717 (Current Evidence)
Model 2	RCT enrolling 200 patients	0.77 [0.44, 1.36]	82	71	717 + 200 = 917
Model 3	RCT enrolling 500 patients	0.77 [0.54, 1.1]	92	80	717 + 500 = 1217
Model 4	RCT enrolling 1000 patients	0.77 [0.6, 0.99]	98	89	717 + 1000 = 1717
Model 5	RCT enrolling 1500 patients	0.77 [0.63, 0.95]	99	93	717 + 1500 = 2217
Model 6	RCT enrolling 2000 patients	0.77 [0.64, 0.92]	100	96	717 + 2000 = 2717
Model 7	RCT enrolling 4000 patients	0.77 [0.68, 0.87]	100	99	717 + 4000 = 4717

Abbreviations: CI, Compatibility (quantile) interval; Pr, Probability of treatment effect; OR, Odds ratio; RCT, Randomized controlled trial

[a] Only considering the invasive mechanical ventilation subgroup

eFigure 4. Prior, Data, and Posterior Distributions in the Normal Conjugate Analysis (Data Mean Equal to 0.77)

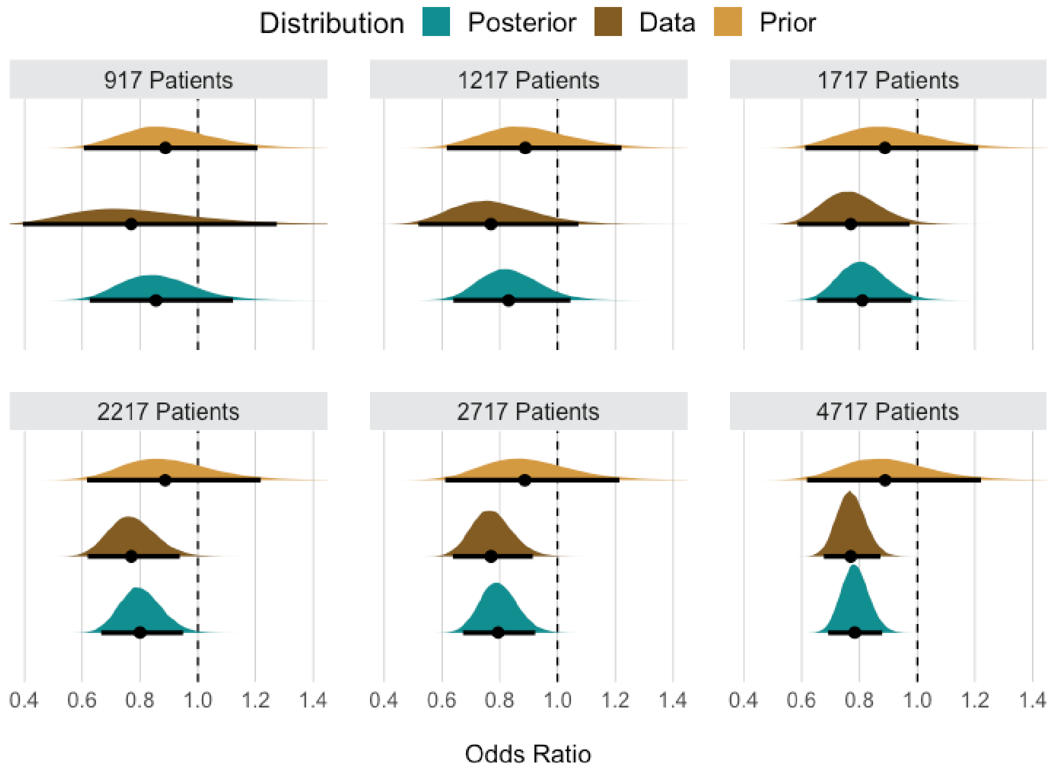

Results from the normal conjugate analyses updating current evidence on invasive mechanical ventilation (used as the Prior) with generated RCTs (used as Data, eTable 5). These analyses yield posterior distributions. Each panel represents a different model, in which the prior distribution is centered at 0.77 odds ratio. The label on top of each panel depicts the number of total patients on invasive mechanical ventilation included in each respective model (current plus generated patients). Point estimates depict the median and interval bars represent the 95% credible intervals for both prior, data (likelihood) and posterior distributions.

eTable 6. Description of the Underlying Generated RCT Used in the Normal Conjugate Analysis (Mean Equal to 1.00)

Model	Generated RCT Characteristics				Number of Patients Included[a]
	Evidence Equivalence	Mean [95% CI]	Pr(OR < 1.0), %	Pr(OR < 0.9), %	
Main Model	-	1 [0.2, 4.99]	50	45	717 (Current Evidence)
Model 2	RCT enrolling 200 patients	1 [0.57, 1.75]	50	36	717 + 200 = 917
Model 3	RCT enrolling 500 patients	1 [0.7, 1.42]	50	28	717 + 500 = 1217
Model 4	RCT enrolling 1000 patients	1 [0.78, 1.28]	50	20	717 + 1000 = 1717
Model 5	RCT enrolling 1500 patients	1 [0.81, 1.23]	50	16	717 + 1500 = 2217
Model 6	RCT enrolling 2000 patients	1 [0.84, 1.19]	50	12	717 + 2000 = 2717
Model 7	RCT enrolling 4000 patients	1 [0.88, 1.13]	50	5	717 + 4000 = 4717

Abbreviations: CI, Compatibility (quantile) interval; Pr, Probability of treatment effect; OR, Odds ratio; RCT, Randomized controlled trial

[a] Only considering the invasive mechanical ventilation subgroup

eFigure 5. Prior, Data, and Posterior Distributions in the Normal Conjugate Analysis (Data Mean Equal to 1.00)

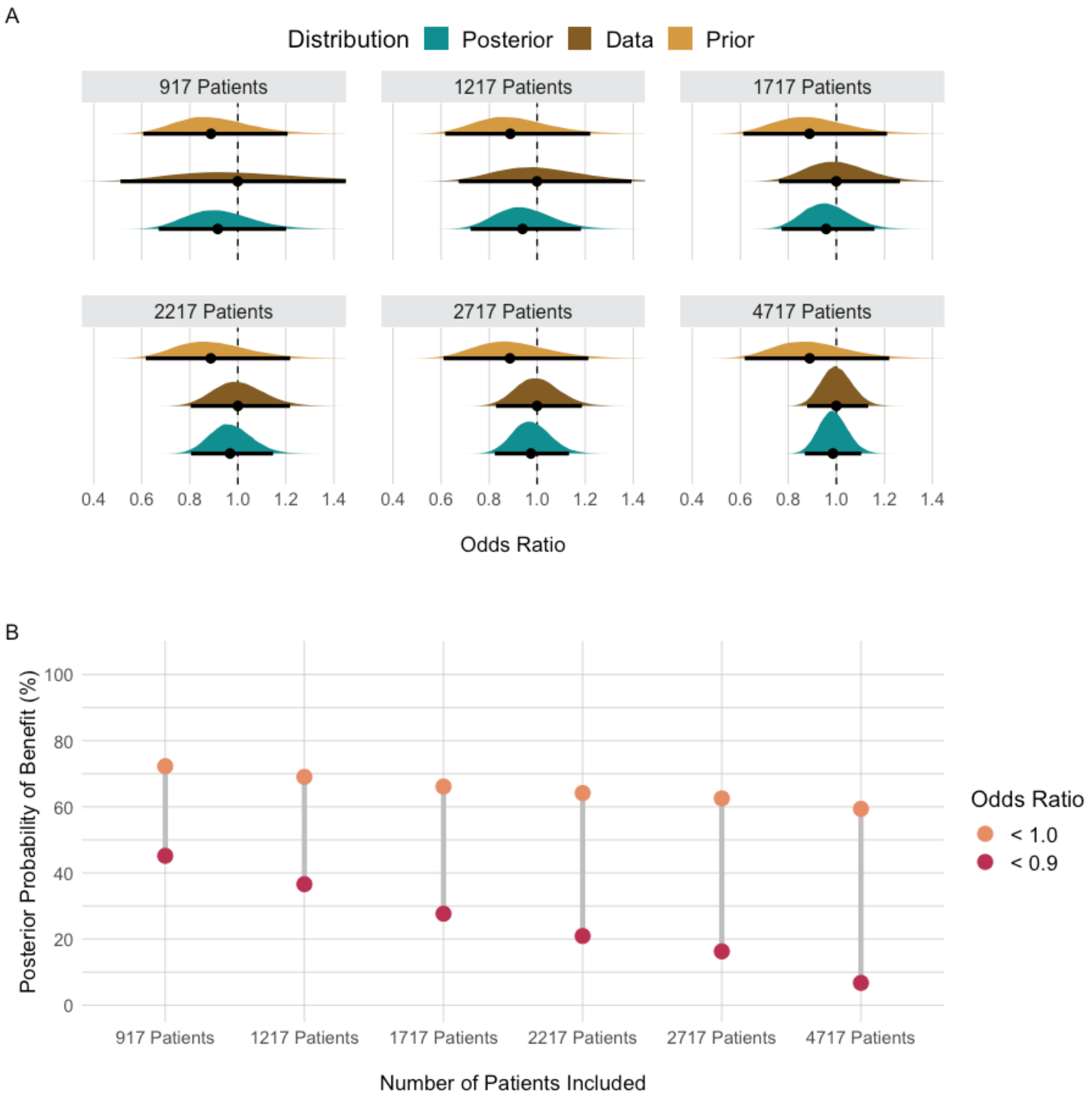

Results from the normal conjugate analyses updating current evidence on invasive mechanical ventilation (used as the Prior) with generated RCTs (used as Data, eTable 6). These analyses yield posterior distributions as depicted on Panel A. In contrast to the results shown in eFigure4, the generated RCTs in these analyses are centered at 1.0 odds ratio. Panel A: Each panel represents a different model. The label on top of each panel depicts the number of total patients on invasive mechanical ventilation included in each respective model (current plus generated patients). Point estimates depict the median and interval bars represent the 95% credible intervals for both prior, data (likelihood) and posterior distributions. Panel B shows the posterior probability of benefit for different thresholds (OR < 1.0 and < 0.9).
